# Multiplexed Genome Editing *via* an RNA Polymerase II Promoter-Driven sgRNA Array in the Diatom *Phaeodactylum tricornutum*: Insights Into the Role of StLDP

**DOI:** 10.3389/fpls.2021.784780

**Published:** 2022-01-04

**Authors:** Yogesh Taparia, Achintya Kumar Dolui, Sammy Boussiba, Inna Khozin-Goldberg

**Affiliations:** Microalgal Biotechnology Laboratory, French Associates Institute for Agriculture and Biotechnology of Drylands, The Jacob Blaustein Institutes for Desert Research, Ben-Gurion University of the Negev, Midreshet Ben-Gurion, Sede Boqer, Israel

**Keywords:** multiplexed genome editing, CRISPR, lipid droplet, Stramenopile-type lipid droplet protein, two-component transcriptional unit, RNA polymerase II promoter, *Phaeodactylum tricornutum*

## Abstract

CRISPR/Cas9-mediated genome editing has been demonstrated in the model diatom *P. tricornutum*, yet the currently available genetic tools do not combine the various advantageous features into a single, easy-to-assemble, modular construct that would allow the multiplexed targeting and creation of marker-free genome-edited lines. In this report, we describe the construction of the first modular two-component transcriptional unit system expressing *Sp*Cas9 from a diatom episome, assembled using the Universal Loop plasmid kit for Golden Gate assembly. We compared the editing efficiency of two constructs with orthogonal promoter-terminator combinations targeting the StLDP gene, encoding the major lipid droplet protein of *P. tricornutum*. Multiplexed targeting of the StLDP gene was confirmed *via* PCR screening, and lines with homozygous deletions were isolated from primary exconjugants. An editing efficiency ranging from 6.7 to 13.8% was observed in the better performing construct. Selected gene-edited lines displayed growth impairment, altered morphology, and the formation of lipid droplets during nutrient-replete growth. Under nitrogen deprivation, oversized lipid droplets were observed; the recovery of cell proliferation and degradation of lipid droplets were impaired after nitrogen replenishment. The results are consistent with the key role played by StLDP in the regulation of lipid droplet size and lipid homeostasis.

## Introduction

Diatoms are found in diverse environments, from polar to tropical, in both marine and freshwater bodies, in aquatic and soil ecosystems. Nearly 20,000 species are estimated to currently exist, with about 12,000 extant species documented in the scientific literature (Guiry, 2012). In ecosystems with high nutrient availability, diatoms rapidly multiply, followed by a collapse in population, displaying typical “boom and bust” cycles. Due to their variety and vast numbers, diatoms are major contributors to the ocean’s carbon, nitrogen, and silicon cycles (Benoiston et al., 2017). Within the marine environment, diatoms constitute a significant portion of phytoplankton, with estimated primary productivity of 45%, and they contribute nearly 20–40% of the global oxygen production. This success and dominance in several environments may be ascribed to their widespread horizontal gene transfer (Vancaester et al., 2020) and their distinct evolutionary origin from red algal secondary endosymbiosis (Armbrust, 2009).

To date, the study of diatom molecular physiology has been dominated by *Phaeodactylum tricornutum*, having a fully sequenced and annotated genome (Bowler et al., 2008) and a growing genetic manipulation toolkit (Apt et al., 1996; Zaslavskaia et al., 2000; Karas et al., 2015; Diner et al., 2016; Bielinski et al., 2017; Serif et al., 2017; Buck et al., 2018; Taparia et al., 2019; Pollak et al., 2020). Moreover, *P. tricornutum* has been used for developing several biotechnological products *via* either heterologous expression (Hempel et al., 2011) or the introduction of entire biosynthetic pathways using synthetic biology tools. Likewise, *P. tricornutum* has been engineered to produce high-value compounds, such as terpenoids (D’Adamo et al., 2018; Fabris et al., 2020) and vanillin (Slattery et al., 2018). *P. tricornutum* is a model oleaginous diatom whose lipid biosynthesis pathways have been intensively investigated. It has been commercially exploited as a source of high-value carotenoids (fucoxanthin) and omega-3 long-chain polyunsaturated fatty acids (LC-PUFA) and other lipids (Falciatore et al., 2020). A large number of genes implicated in the LC-PUFA biosynthesis and storage lipid metabolism have been characterized (Domergue et al., 2005; Hamilton et al., 2015; Haslam et al., 2020), to list a few.

Abiotic stress, such as nutrient deprivation (of nitrogen or phosphorus), triggers the accumulation of storage lipids (Sayanova et al., 2017) in the subcellular organelles, referred to as lipid droplets (LDs). The LD core of *P. tricornutum* is enriched in triacylglycerols (TAG) and enclosed by a monolayer of specific membrane lipids (Lupette et al., 2019). Considering the significance of LD-associated proteins in maintaining LD stability, lipid metabolism, and multifaceted interactions with other cellular compartments, proteomic analyses of isolated LDs have been reported in several studies, with the identification of the *bona fide* LD proteins in *P. tricornutum* and a few more oleaginous diatoms (Yoneda et al., 2016; Lupette et al., 2019; Leyland et al., 2020b). The structural integrity of LDs is deemed to be provided by proteins localized to or associated with the surface of LDs or embedded in the monolayer. However, the functional role of LD proteins in diatoms is yet to be established. One of the most abundant LD proteins, termed as a Stramenopile-type lipid droplet protein (StLDP; Phatr3_J48859), consistently appears in most of the proteomics studies of isolated LDs (Yoneda et al., 2016; Lupette et al., 2019; Leyland et al., 2020b). The amino acid sequence of StLDP features a central hydrophobic domain with conserved proline residues, which is assumed to assist in LD association. GFP-tagged StLDP localizes to LDs in N-deprived *P. tricornutum* (Shemesh, 2015; Yoneda et al., 2018). The overexpression of StLDP caused an increase in neutral lipid production under nitrogen (N) deprivation (Yoneda et al., 2018). Further study of StLDP function is warranted to address its possible role in LD metabolism and lipid homeostasis. However, successful metabolic engineering and functional characterization of candidate genes require efficient genome editing.

Initially, genome editing in *P. tricornutum* was successfully demonstrated *via* TALEN (Serif et al., 2017) and followed by CRISPR/Cas9 (Nymark et al., 2016). CRISPR/Cas9-mediated genome editing is a simple and versatile tool for creating targeted genome modifications and, as a result, has rapidly been adopted for several eukaryotic and prokaryotic species. CRISPR/Cas9 technology is especially valuable for asexual species, such as the model diatom *P. tricornutum*, for which some of the tools used in forward or reverse genetics cannot be applied. Moreover, RNAi for gene expression knock-down is known to be unstable in this diatom (Bielinski et al., 2017). The discovery of *E. coli*-mediated conjugation for the delivery of non-integrating extrachromosomal DNA (diatom episome) to the nucleus of *P. tricornutum* (Karas et al., 2015; Diner et al., 2016) presented an ideal system for delivering CRISPR/Cas9 for genome editing (Sharma et al., 2018; Slattery et al., 2018; Moosburner et al., 2020) without its integration into the nuclear genome. RNP-mediated multiplexed genome editing has also been reported in *P. tricornutum* (Serif et al., 2018). However, in addition to desired genome editing, this system requires the creation of auxotrophs as counterselection markers that require maintenance on media with special supplements. Moreover, most of these reports utilize special equipment for the delivery of gene editing constructs (biolistic gene gun) and expensive screening methods for identifying the resulting genome-editing events (RT-PCR for HRM assay or T7EI assay).

Recently, an episomal multiplexed CRISPR/Cas9 system was shown to use *Sp*Cas9 transcriptionally coupled with *Sh*Ble (selectable marker) to ensure the survival of only those exconjugants that expressed *Sp*Cas9 (Moosburner et al., 2020). Inspired by the successful report of *Sp*Cas9 transcriptionally coupled to the selectable marker, we further improved the system to allow modular assembly and the expression of an sgRNA array from an RNA polymerase II promoter (Mikami et al., 2017; Wang et al., 2018) to episomally express a multiplexed CRISPR/Cas9 system capable of creating genomic lesions that can be detected by a simple colony PCR. In this report, we demonstrate multiplexed targeting of the Stramenopile-type lipid droplet protein (StLDP; Yoneda et al., 2016) gene, leading to a large genomic lesion. Further, we were able to isolate the homozygous deletion lines of the StLDP gene for assessing the effect on phenotypes and traits related to LD biogenesis.

## Materials and Methods

### Media Preparation and *P. tricornutu*m Culture

*P. tricornutum* CCAP1055/1 (Pt 1) cultures were routinely diluted in a 1 × RSE medium for maintenance in an exponential growth phase (Leyland et al., 2020b). *P. tricornutum* were cultured on ¼ × RSE + 1% agar +5% LB as the pre-conjugative and conjugation culture medium following the protocol described in (Diner et al. (2016).

### Assembly of Level-1 Transcriptional Units and the Level-2 Diatom Episome

In the following Level-1-2 and Level-1-3 transcriptional units described, promoter-49202 + terminator-49202 and promoter-H4-1b + terminator FcpA combinations were constantly maintained, and these promoter-terminator combinations occurred orthogonally when assembled in a Level-2 diatom episome multiplexed CRISPR/Cas9 vector. Level-1 and Level-2 constructs were assembled using Golden Gate assembly reactions, as described in Pollak et al. (2019), using a pCA uLoop plasmid kit.

**Figure 1:**
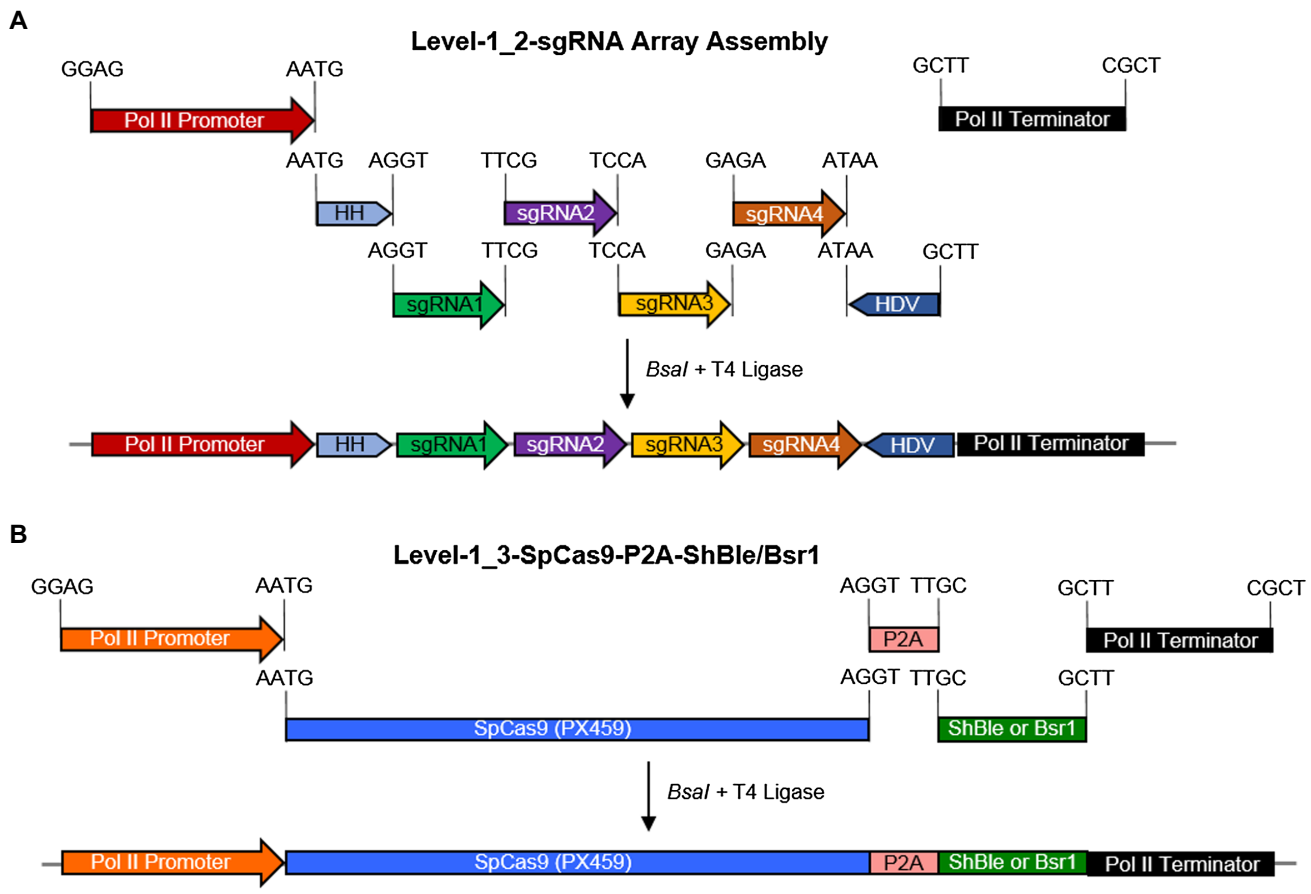
A schematic diagram for the assembly of Level-0 Universal Loop parts into Level-1 transcriptional units and the final assembly of a Level-2 CRISPR/Cas9 diatom episome in the pCA plasmid kit. **(A)** a pCA-Level-1_2-sgRNA array was assembled in a Level-1 Golden Gate reaction with the *BsaI* enzyme and T4 DNA ligase for 30 cycles by combining Level-0 parts and sgRNA fragments with indicated overhangs. **(B)** A pCA-Level-1_3-SpCas9-P2A-*Sh*Ble/*Bsr1* transcriptional unit was assembled in a Level-1 Golden Gate reaction with the *BsaI* enzyme and T4 DNA ligase for 25 cycles by combining Level-0 parts with indicated overhangs. Promoter-terminator combination used for the sgRNA array is not reused for *Sp*Cas9-P2A-*Sh*Ble/*Bsr1* in the same Level-2 episome.

A Level-1_2 sgRNA array expression cassette was assembled using pL0-AC-Pr49202 or pL0-AC-PrH4-1b + pL0-HH-Ribozyme + sgRNA1 + sgRNA2 + sgRNA3 + sgRNA4 + pL0-HDV-Ribozyme + pL0-EF-Tr49202 or pL0-EF-TrFcpA (Figure 1A). Each sgRNA spacer fragment consisted of a complemetary forward and a reverse oligo, which were annealed and extended in a PCR reaction, of the form sgRNA-F aa**ggtct**caE1(N)_20_GTTTTAGAGCTAGAAATAGCAAGTTAAAATAAGGCTAGTCCGTTATC and POS-R aa**ggtct**caE2aaAAAAGCACCGACTCGGTGCCACTTTTTCAAGTTGATAACGGACTAGCCTTATTTTAACTTGCTAT, where bold-face alphabets are the *BsaI* restriction site, E1 and E2 are unique Golden Gate overhangs (https://ggtools.neb.com/getset/run.cgi (Potapov et al., 2018; Pryor et al., 2020), (N)20 is a 20-bp genomic DNA targeting sequence selected from the CRISPOR web-tool (Haeussler et al., 2016; Concordet and Haeussler, 2018), and underlined sequences are complementary bases for annealing and extension in a PCR reaction. Assembly of a Level-1-2 sgRNA array was evaluated by PCR using HH-F (CCGTGAGGACGAAACGAGT) and HDV-R (CCGAAGCATGTTGCCCAGC) primers. Details of the gRNA sequences targeting the StLDP coding sequence are listed in Table 1.

**Table 1:** List of targets used for episome-mediated multiplexed editing of the StLDP gene.

Target	Cleavage position ^a^-Strand_gRNA sequence	CFD score^b^ (0–100)	Predicted efficiency^c^ (0–100)	Off-target^d^ sites
StLDP-1	131-R_GCGACTGGGTAAGTCGTACG	100	74	0
StLDP-2	454-R_TCTGGCACTACAGCGGACGA	100	69	0
StLDP-3	664-F_TTGCCATTTTCAATTCACAA	100	56	0
StLDP-4	837-F_TTGTACGCGAGCACGAACGC	100	70	0

a *Counted from ATG as first base*.

b *Cutting frequency determination (CFD) specificity score. A higher score correlates with a lower off-target effect in the genome (Doench et al., 2016)*.

c *A higher score predicts a greater likelihood of cleavage for the given sequence (Doench et al., 2016)*.

d *Number of off-target sites in the genome for a mismatch within the guide sequence (Concordet and Haeussler, 2018)*.

A Level-1_3 SpCas9 transcriptional unit was assembled using pL0-AC-Pr49202 or pL0-AC-PrH4-1b + pL0-CD-SpCas9(PX459) + pL0-DB5-P2A + pL0-B5E-ShBle (or pL0-B5E-Bsr1) + pL0-EF-Tr49202 or pL0-EF-TrFcpA (Figure 1B). The SpCas9 sequence was amplified from Addgene plasmid #62988 (Ran et al., 2013). Level-1_1 consisted of a *StuI* + *PvuII* digested and religated episome to delete most of the *Sh*Ble cassette, and Level-1_4 consisted of a 24-bp spacer. Annotated nucleotide sequences for each part described above can be found in Supplementary Data Sheet 2.

The Level-2 diatom episome expression plasmid was constructed *via* a one-pot Golden Gate assembly reaction and transformed into NEB® Stable chemically competent *E. coli* and incubated for 20 h at 30°C. Individual *E. coli* colonies were patched on LB agar + Spectinomycin (30 mg/L) plates. An on-colony PCR of patched *E. coli* colonies using HH-F 5′-CCGTGAGGACGAAACGAGT-3′ and HDV-R 5′-CCGAAGCATGTTGCCCAGC-3′ primers was performed to confirm the integrity of the sgRNA array prior to inoculating a culture for plasmid purification. All liquid cultures for *E. coli* (plasmid isolation and conjugation) were inoculated in LB24 medium (Wood et al., 2017) and grown at 30°C with appropriate antibiotics and shaken at 225 rpm.

### Creation of a Conjugative *E. coli* Strain for Delivery of the Diatom Episome

NEB® Stable *E. coli*, transformed with pTA-Mob (Strand et al., 2014), served as a conjugative *E. coli* strain for delivery of a Level-2 expression episome to *P. tricornutum*. Chemically competent NEB® Stable *E. coli*, containing pTA-Mob, was transformed with a Level-2 diatom episome and selected on LB agar plates containing Gentamycin (20 mg/L) + Spectinomycin (30 mg/L) overnight at 30°C. Resultant *E. coli* colonies were patched and rescreened with PCR primers HH-F and HDV-R to ensure the stability of the diatom episome.

### Conjugation and Selection

PCR-verified *E. coli* colonies were used for conjugation as described in Diner et al. (2016). Post-conjugation, *P. tricornutum* cells were selected on ¼ × RSE-agar plates containing Zeocin (100 mg/L) and Kanamycin (50 mg/L).

### Detection of Genome-Edited Strains

Post-selection, exconjugant *P. tricornutum* colonies were screened via colony PCR using the primers INF-LD7-F (GCACATCGGGCTCGGGGTACCCTTCTTCGAGCAATCCGAA) + INF-LD7-R (TAATCATTTACTTAATTAGCTTGCAGGAACAAGCATG). Underlined sequences complementary to the StLDP coding sequence (Phatr3_J48859) were used to calculate the annealing temperature. An amplification product of 1403 bp indicated a lack of gene editing, and a 706 bp or lower DNA fragment indicated multiplexed genome editing.

### Isolation of Homozygous Genome-Edited StLDP Lines

Putative chimeric/heterozygous exconjugants, with unedited and edited PCR bands of nearly equal intensity, were diluted and replated to obtain single colonies. These were patched, and a colony PCR was performed to identify homozygous genome-edited StLDP lines. One of the lines identified as a homozygous genome-edited *StLDP*-KO line was confirmed by Sanger sequencing (Supplementary Figure 1).

### *P. tricornutum* Cultivation and Determination of Growth Parameters

Wild-type and homozygous genome-edited lines were cultivated in an incubator shaker at 22°C and a shaking speed of 120 rpm under a light intensity of 50 μmol photon m^−2^ s^−1^, using a full-strength RSE medium. For the nitrogen deprivation experiments, cells from nutrient-replete cultures were harvested by centrifugation, washed in an N-free RSE medium, and resuspended in an N-free RSE medium at an initial cell density of 5 × 10^6^ cell/ml. Cells were counted using an automated Luna cell counter (BioCat GmbH).

### Fatty Acid Analysis

Fatty acid composition and content were determined as FA methyl esters (FAMEs), following the direct transmethylation of freeze-dried cell pellets and triacylglycerols, isolated from total lipid extracts. Samples placed in glass vials were transmethylated with 2% (v/v) sulfuric acid (H_2_SO_4_) in anhydrous methanol at 80°C for 1.5 h under an argon atmosphere with continuous stirring. Pentadecanoic acid (C_15_:0; Sigma-Aldrich, United States; 0.5 mg/ml in stock solution) was added as an internal standard. The transmethylation reaction was terminated by cooling to room temperature and the addition of 1 ml of H_2_O. FAMEs were extracted with hexane and quantified on a Trace GC Ultra (Thermo, Italy) equipped with an FID and a programmed temperature vaporizer (PTV) injector. The PTV injector was programmed to increase the temperature from 40°C at the time of injection to 300°C at sample transfer. The separation was achieved on a fused silica capillary column (SUPELCOWAX 10, Sigma-Aldrich, United States, 30 m × 0.32 mm) using the following oven temperature program: 1 min at 130°C, followed by a linear gradient to 220°C, and finally 10 min isocratic at 220°C. Helium was used as the carrier gas at a flow rate of 2.5 ml min^−1^. The detector temperature was set at 280°C. FAMEs were identified by co-chromatography with commercial standards (Sigma-Aldrich, United States).

### Lipid Extraction and Triacylglycerol Quantification

Freeze-dried cell pellets (150 × 10^6^ cells) were treated with isopropanol, pre-heated to 80°C, for 10 min under an argon atmosphere. Isopropanol was removed and replaced with a mixture of methanol: chloroform (2:1, v/v). Samples were mixed for 1 h and centrifuged, and the solvent was collected and mixed with isopropanol. DDW was added to form two phases; the bottom phase was collected and evaporated under a stream of N_2_ flow.

Triacylglycerols were separated by one-dimensional TLC on silica gel plates (Silica Gel 60, 10 × 10 cm, 0.25 mm thickness, Merck, Germany), using a solvent system of petroleum ether: diethyl ether: acetic acid (70:30:1, v/v/v). Lipid spots were visualized with iodine vapors, scraped from the plates, and transmethylated as described above.

### Microscopy

Cells were observed under a Zeiss microscope (Carl Zeiss, Göttingen, Germany) equipped with an AxioCam digital camera, differential interference contrast (DIC) Nomarski optics, and epifluorescence. Lipid droplets were visualized using Nile Red staining: 1 μl of Nile Red (Sigma-Aldrich) solution in DMSO (100 μg/ml) was added to 10 μl of the sample, mixed by pipetting, and observed under a Filter Set 16 with a bandpass excitation at 485/20 nm and a long-pass emission beyond 515 nm.

## Results

### Golden Gate Assembly Based a uLoop System Enables the Construction of a Highly Modular Genome-Editing Episome With Multiplexed sgRNA Capability

Golden Gate assembly combines a restriction-ligation cloning method into a one-pot reaction, which allows a seamless combination of interchangeable modules, thus achieving a combinatorial synthesis of expression vectors in parallel. Using an expanded syntax of the uLoop system, four sgRNA targets for the StLDP gene (Phatr3_J48859) were seamlessly assembled into a Level-1 transcriptional unit (Figure 1A). Similarly, a *Sp*Cas9 coding sequence was translationally coupled to the *Sh*Ble/*Bsr1* selectable marker *via* a P2A peptide with the facility to exchange promoter and terminator sequences as demanded by experiments or for future optimization (Figure 1B).

### Stable Assembly and Delivery of a Level-2 Diatom Episome With Tandem sgRNA Repeats

While a multiplexed sgRNA transcriptional unit can be assembled into a Level-1_2 construct using routine cloning strains, such as NEB® Turbo, assembly with Level-1_3-*Sp*Cas9 into the final Level-2 expression episome fails without the use of a special *E. coli* strain, such as NEB® Stable, which is capable of maintaining DNA with multiple repeats. The use of LB24 broth (Wood et al., 2017) for culturing NEB® Stable *E. coli* at 30°C and 225 rpm ensured a comparable growth rate to EPI300™ *E. coli* grown at 37°C and 225 rpm, as used routinely for the conjugative delivery of the episome to *P. tricornutum*. To maintain episome stability, while culturing NEB® stable cells, LB agar plates and broth were incubated at 30°C. Slower growth in a liquid medium at 30°C was compensated by using a rationally balanced medium, such as LB24 [35].

### Use of RNA Polymerase II Promoters Allows the Multiplexed Expression of an sgRNA Array

A tandem array of sgRNA targets, flanked by Hammer Head and Hepatitis Delta Virus ribozymes, was expressed as a single transcript using an RNA Pol-II promoter, precluding the use of multiple Pol-III promoter-terminator cassettes for each target and leading to a compact and efficient assembly of the CRISPR/Cas9 editing episome. Interspersing 6 bp filler sequences (5′-AA-3′ + 4 bp-GG overhang) between each sgRNA target allowed the endogenous ribonuclease processing of the tandem sgRNA array into active Cas9-RNP for genome editing.

### Evaluation of Genome-Editing Events and Their Frequency With Two Constructs With Orthogonal RNA Polymerase II Promoters

**Figure 2:**
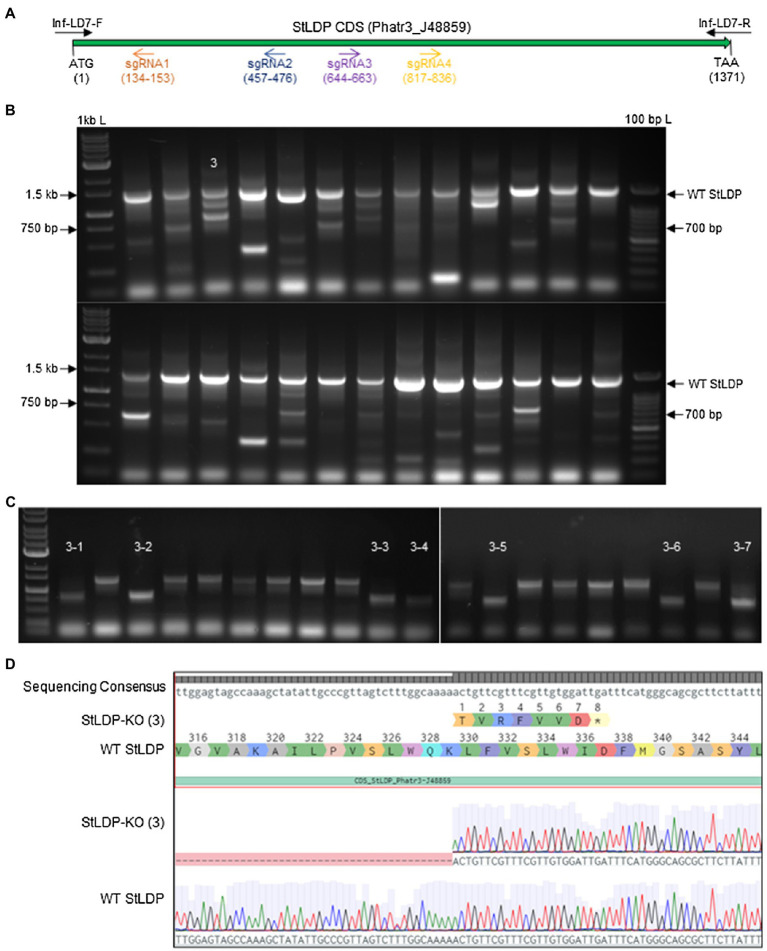
PCR screening for gene editing in primary exconjugants in *P. tricornutum* Pt1 (CCAP1055/1). **(A)** A schematic of the StLDP gene (Phatr3_J48859), indicating positions of sgRNA-guided cleavage sites. **(B)** Twenty-six primary exconjugants with heterozygous or chimeric deletions in the StLDP gene are shown. Wt *StLDP*, amplified with INF-StLDP-F and INF-StLDP-R primers (Materials and Methods), produces a 1,400-bp product; amplicon below 700 bp indicates a deletion resulting from the multiplexed targeting of all four sgRNA. 1 kb L = GD 1 kb DNA Ladder RTU from GeneDireX Inc.; 100 bp L = 100 bp DNA Ladder RTU from GeneDireX Inc. **(C)** PCR screening of single colonies from primary exconjugant StLDP (3) to identify lines with homozygous deletion. **(D)** Partial sanger sequencing of the StLDP gene in primary exconjugant StLDP (3).

Using four targets against the coding sequence of the StLDP gene of *P. tricornutum* (Table 1), we created a ~ 500 bp lesion detected by a simple on-colony PCR-based screening strategy (Figure 2). A direct on-colony PCR of *P. tricornutum* exconjugants allowed the rapid detection of genome-editing events for further study and evaluation (Figure 2B). The majority of edited events resulted from the construct in which *Sp*Cas9(PX459) and *Sh*Ble expression was driven by Pr49202-Tr49202, and the sgRNA array was transcribed by the PrH41b-TrFcpA promoter-terminator combination, which displayed editing at a frequency of 6.7–13.8% (Table 2). The sequences of the promoter and terminator combinations can be found in (Pollak et al., 2020). A PCR screening revealed that most primary exconjugants were either chimeric or heterozygous mutants (Figure 2B). The bands detected between the WT StLDP (~1,400 bp) and 700 bp (product of all four sgRNAs simultaneous deletion) are most likely deletions resulting from two or three sgRNAs.

**Table 2:** Editing efficiency of two episome constructs with orthogonal RNA polymerase II promoters driving gRNA and Cas9 expression to detect deletions in the StLDP gene.

Promoter-terminator (sgRNA array/Cas9-P2A-ShBle)	Repetition	TotaleExconjugants	Edited events detected by PCR	Efficiency (%)
	1	80	9	11.3
PrH4_1b-TrFcpA / Pr49202-Tr49202	2	167	23	13.8
	3	120	8	6.7
	1	56	1	1.8
Pr49202-Tr49202/PrH4_1b-TrFcpA	2	38	1	2.6
	3	40	5	12.5

### Isolation of Homozygous Deletion StLDP Mutants

PCR-screened primary exconjugants, which displayed gene editing and the resultant PCR amplicons that were equal in intensity to the wild-type *StLDP* sequence, were diluted and replated. Lines with homozygous deletion were confirmed *via* PCR screening from the single colonies emerging from the replating (Figure 2C). Sanger sequencing from one of the lines, designated *StLDP*-KO (3), further confirmed the line to be a homozygous deletion event (Supplementary Figure 1; Figure 2). Zeocin selection pressure was maintained till the isolation of single colonies with a 500 bp homozygous deletion. In order to eliminate the background effect of Cas9 and conjugation, the lines were diluted several times under nonselective conditions to allow the CRISPR episome to be cured (Karas et al., 2015). These gene-edited lines (additionally confirmed by PCR on aliquots of liquid cultures; Supplementary Figure 2) free of the CRISPR/Cas9 episome were then compared to the WT under specified experimental conditions to evaluate the effect of disruption of the StLDP gene on LD formation.

### Gene-Edited Lines Display Growth Phenotype and Accumulate Lipid Droplets in Replete Conditions

**Figure 3:**
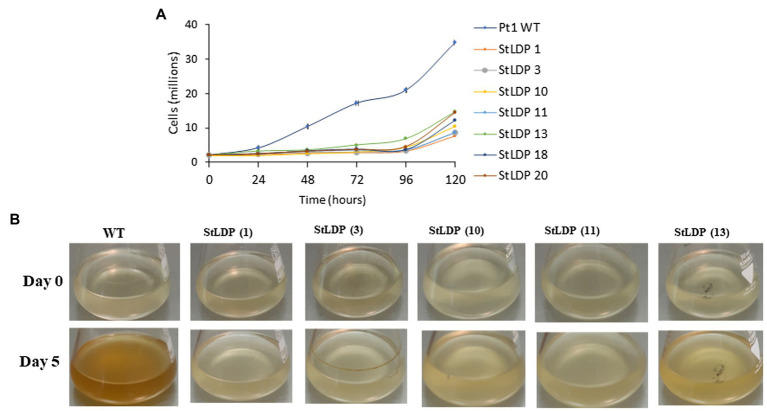
Growth of wild-type Pt1 and *StLDP*-KO lines in the nutrient-replete medium. **(A)** Growth of the cultures monitored by cell counting. **(B)** Images of *P. tricornutum* cultures, representing relative growth from day 0 to 5 days of cultivation in the nutrient-replete medium. The initial cell density −2 × 10^6^ cells/ml. Wild-type and *StLDP*-KO lines were cultivated in an incubator shaker with a CO_2_-enriched atmosphere (100 ml/min) at 22°C with a shaking speed of 120 rpm under a light intensity of 50 μmol photon m^−2^ s^−1^.

**Figure 4:**
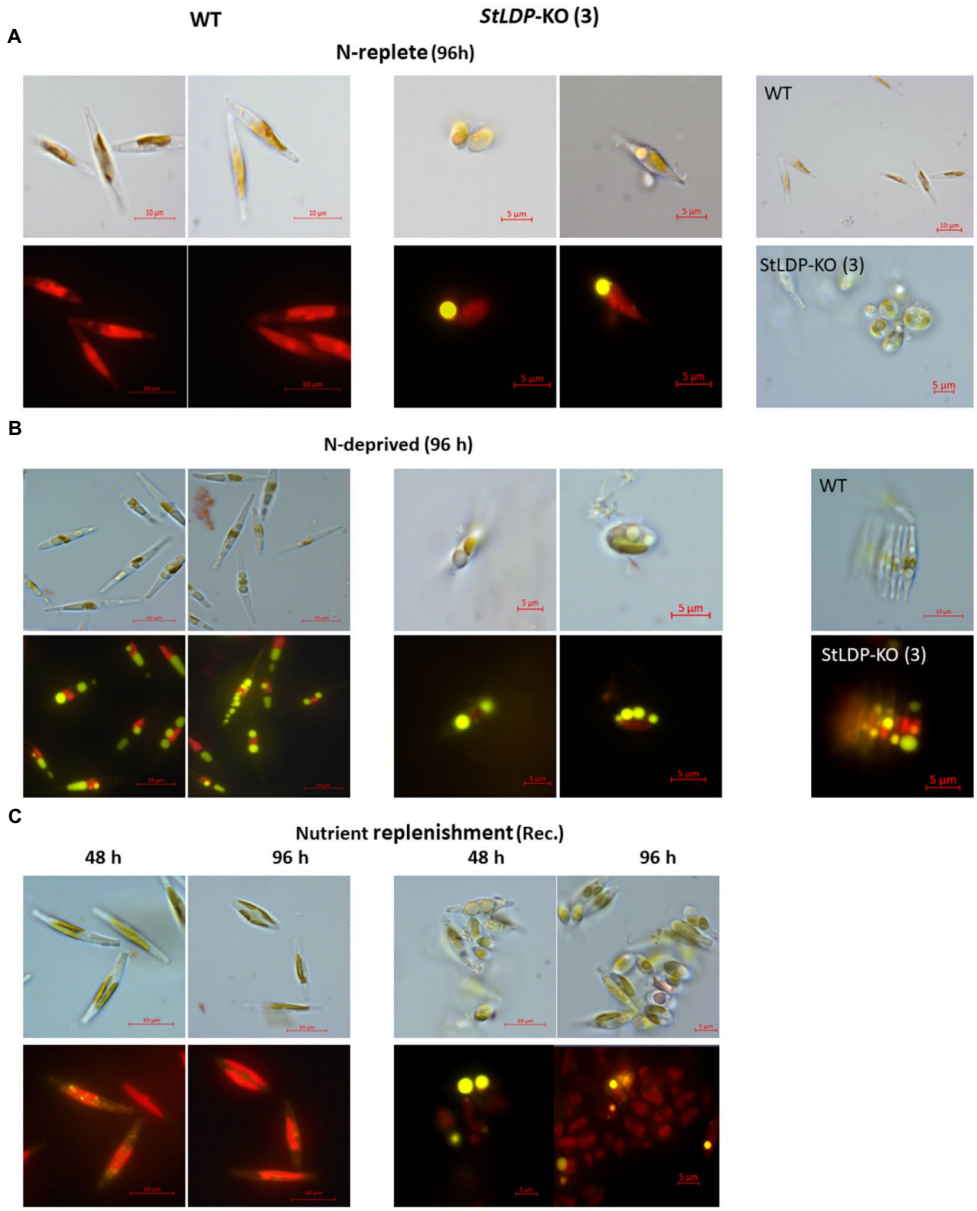
Micrographs of Pt1 wild-type and *StLDP*-KO (3) cells under N-replete conditions **(A)**, N deprivation **(B)**, and following nutrient replenishment **(C)**. DIC micrographs and fluorescence micrographs of Nile Red stained cells (×1,000 magnification) are shown for each condition (indicated). Lipid droplets are visible as bright yellow fluorescent bodies. Pt1 wild-type and *StLDP*-KO (3) lines were cultivated in an incubator shaker with a CO_2_-enriched atmosphere (100 ml/min) at 22°C with shaking speed of 120 rpm under a light intensity of 50 μmol photon m^−2^ s^−1^.

In this work, we report the initial characterization of the gene-edited lines in terms of growth, morphology, and fatty acid production under N-replete and N-deprived conditions. Seven transgenic *StLDP*-KO lines were monitored in the nutrient-replete medium for characterizing their growth performance (cell concentration) and microscopic analysis. All the examined gene-edited lines exhibited impaired growth and bleached phenotypes compared with the wild-type Pt1 (Figures 3A,B). All seven *StLDP*-KO lines exhibited virtually the same growth inhibition relative to the wild type (Figure 3A). Furthermore, light microscopy screening revealed that *StLDP*-KO lines formed LDs during the growth in the nutrient-replete medium. For a detailed microscopic analysis, we selected line *StLDP*-KO (3) with the 500 bp deletion confirmed by sequencing (Supplementary Figure 1). The morphology of *StLDP*-KO (3) cells grown in the replete medium differed markedly from the wild-type cells, dominated by fusiform cells. Three morphotypes of fusiform, circular, and oval cells were observed in the *StLDP*-KO (3) culture. In the wild-type cells grown in the N-replete medium, LDs, visualized by Nile Red staining, were either tiny or absent (Figure 4A), while *StLDP*-KO (3) cells produced large LDs (Figure 4A). Fusiform cells had one to two LDs with a diameter of 1.5–2 μm; however, multiple LDs were occasionally observed (Figure 4A). Oval and circular cells with a single LD were most abundant in the culture of *StLDP*-KO (3); these cells were prone to aggregation and the formation of stable clumps (Figure 4A). Because aggregation of oval and circular cells hindered cell counting and staining the LDs with Nile Red, intensive vortexing was required before the analysis.

Following the transfer to the N-deprived medium (Figure 4B), the cells of *StLDP*-KO (3) with pre-formed LDs did not display substantial morphological changes compared with the cells in nutrient-replete medium; furthermore, cell clumping increased. Circular LDs occupied most of the cell volume, while the plastid and other compartments seemed to be substantially abridged. Cell clumping decreased after nutrient replenishment (Figure 4C); however, clumps were observed after 96 h of recovery. Within 48 h following nutrient resupply, LDs disappeared from wild-type cells, whereas large, circular LDs were observed in the *StLDP*-KO (3) cells.

### Gene-Edited Lines Accumulate TAG in Replete Conditions

**Figure 5:**
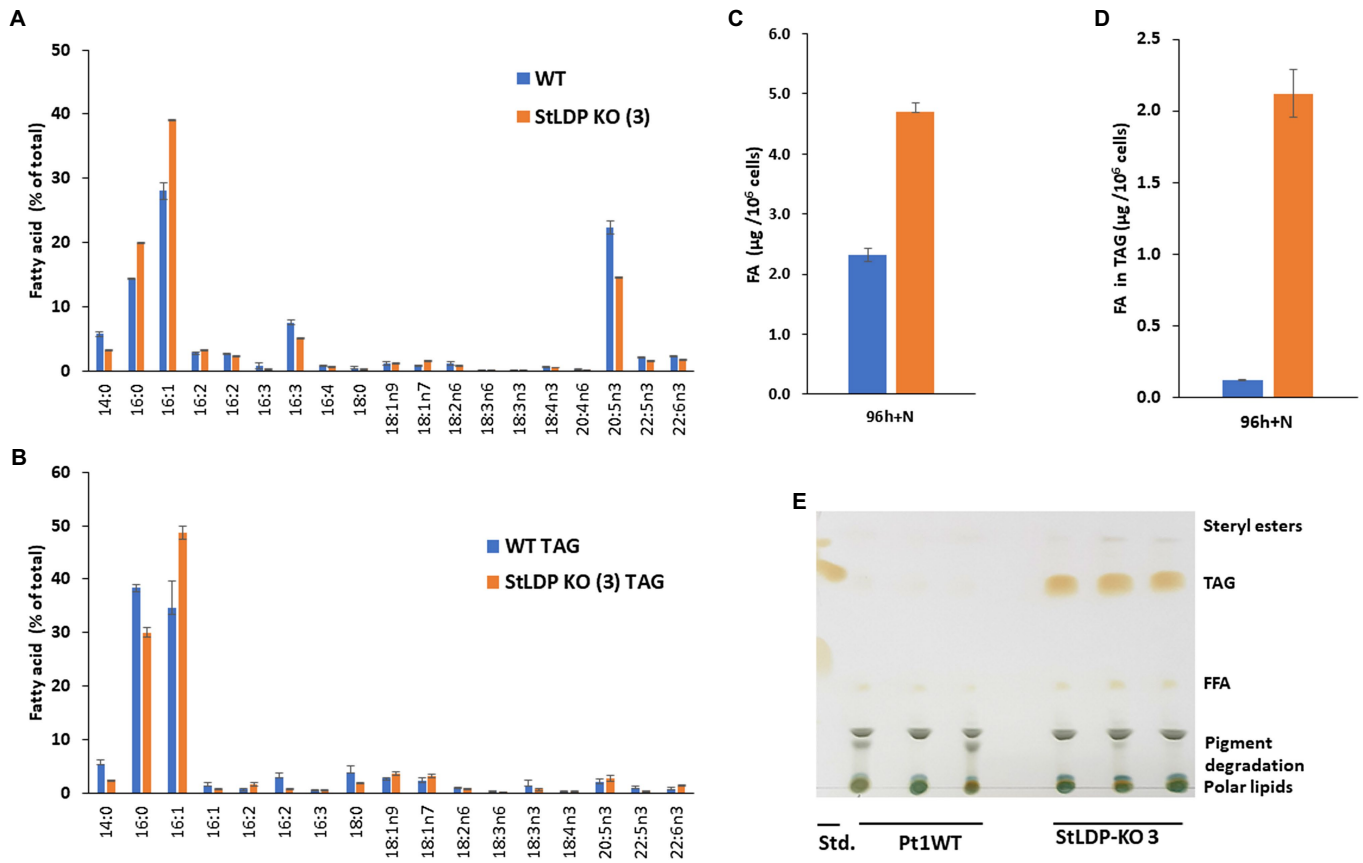
Fatty acid analysis of wild-type Pt1 and *StLDP*-KO (3) under nutrient-replete conditions by gas chromatography (GC). **(A)** Relative abundance of total fatty acids (%) after 96 h of cultivation in a nutrient-replete medium. **(B)** Total fatty acid content per 10^6^ cells. **(C)** Relative abundance of fatty acids in TAG (%). **(D)** Fatty acid content in TAG per 10^6^ cells. **(E)** Separation of neutral lipids by TLC in a solvent system used for TAG isolation.

To examine whether and how the lesion in the *StLDP* gene affected the production and the relative abundance of fatty acids (% of total) under N-replete and N-deprived conditions and following nutrient replenishment, a FAMEs analysis was performed. Under nutrient-replete conditions, the fatty acid profile of the *StLDP*-KO (3) line showed a reduction in the proportion of the major omega-3 LC-PUFA, eicosapentaenoic acid, 20:5*n*3, as well as a noticeable increase in the proportion of palmitoleic acid (16:1*n*7) compared with the wild type (Figure 5A). The cells of the *StLDP*-KO (3) accumulated *ca*. 2.5-fold more fatty acids compared with the wild type under nutrient replete conditions (Figure 5C).

Since the *StLDP*-KO (3) line exhibited LD formation in replete conditions, we isolated TAG from the total lipid extract by one-dimensional TLC and subsequently quantified acyl groups accumulated in TAG as FAMEs. As was evident from the imaging of the TLC plate after exposure to iodine vapors, the *StLDP*-KO (3) accumulated TAG under replete conditions, while TAG formation was minute in the wild-type cells (Figures 5D,E). Quantification of fatty acids confirmed a substantial accumulation of TAG in the *StLDP*-KO (3) cells under nutrient-replete conditions. The main differences in the fatty acid profile of TAG comprised the increased percentage of 16:1 and a decrease of 14:0 and 16:0 compared with the TAG of the wild type (Figure 5B).

**Figure 6:**
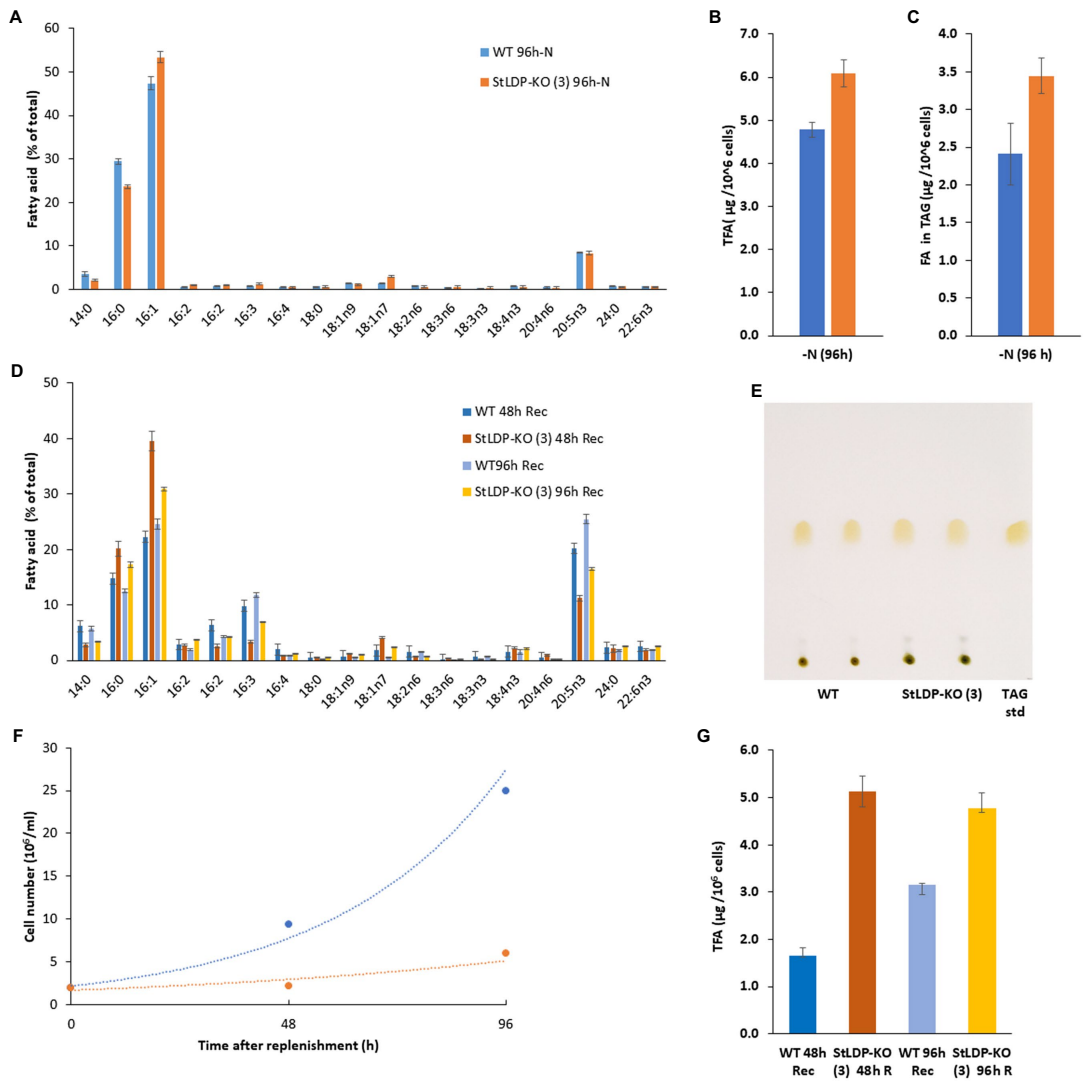
Comparative analysis of wild-type Pt1 and *StLDP*-KO (3) under N deprivation and following nutrient replenishment. **(A)** Relative abundance of fatty acids (% of total) after 96 h of cultivation in the N-deprived medium. **(B)** Total fatty acid content (μg/10^6^ cells) after 96h of N deprivation. **(C)** Fatty acid content in TAG (μg/10^6^ cells) after 96h of N deprivation. The initial cell density −5 × 10^6^ cells/ml. **(D)** Relative abundance of fatty acids (% of total) during recovery from N-deprivation. **(E)** Isolation of TAG from N -deprived cells by TLC: total lipids were loaded based on the equal cell number. **(F)** Cell concentration dynamics after nutrient replenishment. The initial cell density −2 × 10^6^ cells/ml. **(G)** Relative abundance of fatty acids (% of total) 48 and 96 h after nutrient replenishment.

Under N deprivation, the main difference in the fatty acid profile comprised a decrease in the proportion of 16:0 with a concomitant increase in 16:1*n*7 (Figure 6A). In the course of nitrogen deprivation, the TFA content markedly increased in the wild type to 4.8 μg/10^6^ cells (Figure 6B). In contrast, there was a relatively mild increase in the *StLDP*-KO (3) line (to *ca*. 6.0 μg/10^6^ cells), compared with the N-replete cells (Figure 6B). TLC analysis performed to quantify TAG produced under N deprivation indicated that TAG increased less in the KO line than in the wild type (Figure 6C). Yet, the TAG content was higher in the *StLDP*-KO (3) cells. Following the resupply of nutrients to N-deprived cells, in line with the growth (Figure 6A) and microscopy data (Figure 4C), the growth recovery and the decrease in fatty acid content (per 10^6^ cells) was impeded in *StLDP*-KO (3). In the wild type, the content of total fatty acids drastically decreased within 48 h, from 5 to 1.5 μg/10^6^ cells, followed by an increase to ~3.0 μg/10^6^ cells over the next 48 h (Figure 6B). In contrast, the total fatty acid content remained substantially higher in the *StLDP*-KO (3), but there was a steady, though relatively mild, decrease in the *StLDP*-KO (3). The fatty acid profile comprised ~40% of 16:1 and 20% of 16:0 in the *StLDP*-KO after 48 h of nutrient resupply (Figure 6D). In the wild type, these fatty acids comprised ~20 and 15%, respectively after 48 h of nutrient resupply. Since 16:0 and 16:1 are the major constituents of TAG in *P. tricornutum* (Figure 5E), this result may corroborate a delayed hydrolysis of TAG and LD breakdown in *StLDP*-KO (3) cells. Another indication of the impaired recovery of *StLDP*-KO (3) cells from N deprivation was the reduced proportion of the EPA of total fatty acids. By 96 h, the effect was moderate, as indicated by an increase in the percentage of two PUFAs, 16:3 and 20:5*n*3, associated with plastid membrane lipids.

## Discussion

Although several incremental improvements have been achieved in deploying CRISPR/Cas9 for genome editing in *P. tricornutum* (Nymark et al., 2016; Kroth et al., 2018; Serif et al., 2018; Sharma et al., 2018; Stukenberg et al., 2018; Moosburner et al., 2020), none of these reports, to date, have incorporated the latest developments in multiplexed genome targeting as a modular system allowing rapid construct assembly for creating large genomic lesions that can be easily detected by PCR. Here, we described a scheme for modular assembly of a multiplexed genome-editing episome, based on Universal Loop assembly (Pollak, 2019; Pollak et al., 2019, 2020), to enable a simple PCR-based screening of edited lines, free of selection markers.

Multiplexed genome targeting allows the simultaneous editing of two or more loci or the creation of a large deletion that can be detected easily by routine PCR screening. Several strategies have been described in other model systems for multiplexing sgRNA expression from constructs that include stacked RNA polymerase III sgRNA expression cassettes (Moosburner et al., 2020), tRNA-sgRNA arrays expressed from RNA polymerase III promoters (Xie et al., 2015), and ribozyme-gRNA-ribozyme (RGR) arrays transcribed from RNA polymerase II/III promoters (Gao and Zhao, 2014).

The majority of multiplexed sgRNA expression systems utilize snRNA/ U6/U3 (RNA polymerase III) promoters, which are inherently inefficient at transcribing a long array of sgRNAs, typically interspersed with tRNA or ribozymes or Csy4-28 bp linkers (Hsieh-Feng and Yang, 2019). This limitation can be overcome by using RNA polymerase II promoters, which can easily transcribe long sgRNA arrays containing ribozymes for efficient cleavage of the array-transcript into mature sgRNAs. An efficient TCTU system for multiplexed gene editing in rice utilizing an RNA polymerase II promoter for transcribing an RGR array, was recently described, allowing simultaneous editing in three targets (Wang et al., 2018). Furthermore, in rice (Mikami et al., 2017), demonstrated that ribozymes are optional for processing the SpCas9-sgRNA transcript into functional RNP due to the presence of endogenous ribonucleases.

In this study, we combined the use of an RNA polymerase II-based TCTU approach to CRISPR/Cas9 from (Wang et al., 2018), the use of single HH and HDV ribozymes for the entire four target sgRNA array from (Gao and Zhao, 2014), an endogenous sgRNA processing capability from (Mikami et al., 2017), and a selectable marker transcriptionally coupled to SpCas9(PX459) expression (Moosburner et al., 2020) into an efficient, compact, episomally expressed CRISPR/Cas9 genome-editing system with multiplexed capability (Figure 1). Although the editing efficiency reported here is lower than that reported in other studies, our estimates are more conservative due to the fact that they are based on an overall observation of a large genic deletion rather than on an sgRNA-by-sgRNA basis. Moreover, the editing detection strategy used in this report is simple, requiring no special equipment or additional reagents in contrast to the methods, such as HRM or T7EI, used in other reports.

Recently, a Universal Loop kit (Pollak et al., 2019, 2020), a Golden Gate assembly-based cloning system, was released for several photosynthetic microalgae, including *P. tricornutum*. Such a Golden Gate cloning-based system offers an efficient, one-pot assembly of DNA parts with standardized overhangs, up to 24 parts (Potapov et al., 2018; Pryor et al., 2020). Two strong promoter-terminator combinations, PrH4_1b-TrFcpA (Slattery et al., 2018) and Pr49202-Tr49202 (Bielinski et al., 2017), were selected from the kit to construct a TCTU system. These were orthogonally combined either to transcribe the ribozyme-flanked sgRNA array or to express the SpCas9-P2A-ShBle cassette into two diatom episome constructs for testing editing efficiency. Surprisingly, of the two constructs, in the combination where the promoter H4_1b-terminator FcpA was used for expressing the sgRNA array and the promoter 49,202-terminator 49,202 was used for expressing SpCas9-P2A-*Sh*Ble, the number of exconjugants and gene editing was observed at a greater frequency. Moreover, most of the deletions observed were either chimeric or heterozygous. This highlights the need for testing more promoters, preferably from genes involved in cell division or circadian rhythm. Indeed, a recent study in *Chlamydomonas reinhardtii* reported an increase in homology-directed repair events or non-homologous end-joining events based on the time point within the cell cycle, when circadian-rhythm-synchronized cultures were delivered to Cas9 RNP (Angstenberger et al., 2020). Our study further suggests the feasibility of deploying a single transcriptional unit CRISPR/Cas9 without the use of ribozymes for multiplexed genome targeting. Such improvements in multiplexed genome-targeting systems hold promise for efficient transcriptional modulation or repression *via* CRISPRa and CRISPRi, especially for a model diatom species, such as *P. tricornutum*, allowing a thorough investigation of metabolic pathways and further opening avenues for their engineering.

Delivery of CRISPR/Cas9 has been achieved in *P. tricornutum via* the biolistic transformation of plasmids (Nymark et al., 2016; Sharma et al., 2018) or RNP (Serif et al., 2018) and *E. coli*-mediated conjugation (Sharma et al., 2018; Slattery et al., 2018; Stukenberg et al., 2018; Moosburner et al., 2020). Of all these methods, *E. coli*-mediated conjugation requires no specialized equipment or chemicals to maintain auxotrophs (for RNP counterselection) and can be performed in a high-throughput format. Additionally, the diatom episome can be cured upon the removal of selection pressure. While the assembly of constructs with one or two sgRNA targets is easily achieved, the addition of more targets for multiplexing increases repetitive DNA in plasmids and makes the construct unstable in *E. coli*. In our experiments, a Level-2 multiplex sgRNA episome could only be assembled in NEB® Stable *E. coli* specialized for maintaining plasmids with repetitive DNA. However, the assembly of a Level-1-2 sgRNA transcriptional unit with four targets could easily be achieved with regular *E. coli* strains, such as NEB® Turbo. Similarly, for the *E. coli*-mediated delivery of the multiplexed CRISPR/Cas9 episome, NEB® Stable *E. coli* was transformed with pTA-Mob (Strand et al., 2014) and the Level-2 diatom episome.

Multiplexed genome editing of the StLDP gene successfully created targeted and stable gene knockouts in *P. tricornutum* cells, carrying the genic lesion in the gene encoding StLDP and showing a strong growth impairment. *StLDP*-KO cells adopted altered shapes and morphologies distinct from the typical fusiform cells of the Pt1 wild type, suggesting a possible role of StLDP or the downstream effect of its KO in the cell morphology regulation. Furthermore, oval and circular cells were amply present in cultures of the edited lines, and they formed cell clumps. It is known that *P. tricornutum* cells secrete exopolymeric substances (EPS) with adhesive properties, particularly the oval cells (Willis et al., 2013; Galas et al., 2021). It has also been suggested that oval cells represent a form that is more resilient to stresses, in Galas et al. (2021) and references therein.

Furthermore, the StLDP gene KO resulted in LD formation and TAG accumulation in nitrogen-replete conditions. While the molecular mechanisms of LD formation in the N-replete cells of the gene-edited lines warrant further investigation, we can provide several plausible explanations. We speculate that the ablation of StLDP and the resulting changes in the assemblage of LD-associated proteins may disturb LD turnover in the replete cells followed by the coalescence of forming LDs. Generally diminutive or absent from the replete cells, LDs and the storage lipids sequestered within LDs undergo continuous turnover, as corroborated by knocking down the genes for major TAG lipases in two diatoms (Trentacoste et al., 2013; Barka et al., 2016). The study of the peculiar dynamics of LD formation by *P. tricornutum* strain Pt1 (Jaussaud et al., 2020) revealed a complex mode of LD size control and the lack of LD fusion in the presence of StLDP under N deprivation.

Furthermore, since the nature and composition of major lipid droplet proteins vary among different species, the downstream effect of knockdown/knockout of genes encoding proteins on lipid metabolism might be species- and taxon-dependent (Yoneda et al., 2016). For example, in the green microalga *C. reinhardtii*, RNAi-mediated silencing of *MLDP* led to an increase in the diameter of LDs in N-deprived conditions, without, however, affecting the TAG content (Moellering and Benning, 2010). Cultivation of *StLDP*-KO cells in the N-deprived medium caused detrimental effects on cell morphology (agglomerated circular and oval cells with oversized LDs) and did not cause a substantial increment in the TAG content per cell. Following nutrient replenishment, the recovery of *StLDP*-KO cells was severely impaired. The remobilization of storage lipids in the *StLDP*-KO during recovery was delayed, although not completely impaired, and coincided with at least partial recovery of the membrane systems, as indicated by microscopy and FA profiling (an increase in 16:3 and 20:5). The delay in TAG degradation could be attributed to the fact that the reduced surface area per volume of LDs in StLDP led to decreased TAG hydrolysis by lipases, acting in LD turnover at the LD surface. For example, in *Arabidopsis thaliana*, decreased lipid remobilization was observed when a gene for oleosin 1, a lipid droplet resident protein, was knocked out (Siloto et al., 2006). Consistent with our observation of the formation of oversized LDs in the *StLDP*-KO line, enlarged LDs, which did not cease to coalesce, were observed in the *mldp* lines of *C. reinhardtii* under prolonged N deprivation (Tsai et al., 2015). However, in the *StLDP-KO* line LDs were formed and TAG accumulated in replete cells, indicating a significant impact on TAG turnover. Further, the reduction of MLDP in *C. reinhardtii* caused a delay in TAG breakdown, which is consistent with our results. Therefore, our study indicates the important role played by StLDP in regulating LD size and number in *P. tricornutum* and suggests a possible function in stabilizing the LD surface. A more detailed study of the *StLDP*-KO lines is further required to reveal the role of this major LD protein in *P. tricornutum*.

Lipid droplets can also be degraded by the autophagy-related lipophagy process (Zienkiewicz and Zienkiewicz, 2020). It has recently been shown that the major LD surface protein of the eustigmatophyte *Nannochloropsis oceanica* interacts with the hallmark autophagy protein ATG8 during the autophagic catabolism of LDs (Zienkiewicz et al., 2020). The use of fluorescent-protein-tagged ATG8 helped to reveal contacts between LDs and autophagic structures in *C. reinhardtii* (Tran et al., 2019). A similar mechanism may operate in *P. tricornutum*, as suggested by the presence of the ATG8-interacting motif in StLDP (Leyland et al., 2020a). Therefore, it is possible to speculate that the ablation of StLDP disturbs autophagic degradation of LDs in nutrient-replete conditions, consistent with the augmented formation of LDs in replete cells and the impeded degradation following nutrient replenishment. These suggestions and other possible mechanisms and effects of StLDP ablation on cellular and LD proteomes and lipidomes will be experimentally tested in future research.

## Conclusion

In this work, we have demonstrated the creation of a large homozygous deletion in the StLDP gene *via* a multiplexed CRISPR/Cas9 array expressed from the diatom episome. This report describes the essential cloning steps and *E. coli* strains required for successful accomplishment of multiplexed genome editing. We demonstrate here the feasibility of efficient multiplexed gene editing in a TCTU system *via* the diatom episome. Multiplexed targeting *via* RNA polymerase II promoters greatly reduced the size of the episome construct and overcame the necessity to find new snRNA/RNA polymerase III promoters for target stacking in a construct, especially for a recent model system. *E. coli*-mediated episome delivery of CRISPR/Cas9 to diatoms can be cured of exconjugants creating non-GM edited lines, which are desirable in commercial cultivation. Moreover, the US has recently deregulated gene editing in crops for creating single base changes or several base-pair deletions that can be achieved by traditional breeding practices (Stokstad, 2020). Our work also suggests that sgRNA arrays can be efficiently processed into functional sgRNA by endogenous ribonucleases in *P. tricornutum*, allowing the accommodation of more sgRNA targets instead of ribonucleases. Deploying four targets may provide a greater chance of ensuring generation of mutants with large deletion within a given genomic region. Moreover, four targets may be further used for creating double mutants in a single experiment. We hope that the Level-0 parts generated in this study (Supplementary Material) will be useful within the *P. tricornutum* community for further optimization of CRISPR/Cas9-mediated gene editing. The effectiveness of the method was exemplified by creating mutants of the major lipid droplet protein StLDP, allowing novel insights into its role in LD metabolism.

## Data Availability Statement

All sequences and data relevant to reproducing this work has been included in the article and its **Supplementary Material**.

## Author Contributions

YT and IK-G: conceptualization. YT and AKD: methodology. YT, AKD, and IK-G: formal analysis and writing – original draft preparation. IK-G: writing – review and editing and funding acquisition. IK-G and SB: supervision. All authors contributed to the article and approved the submitted version.

## Funding

This research was supported by the Israel Science Foundation (grant no. 1958/18).

## Conflict of Interest

The authors declare that the research was conducted in the absence of any commercial or financial relationships that could be construed as a potential conflict of interest.

## Publisher’s Note

All claims expressed in this article are solely those of the authors and do not necessarily represent those of their affiliated organizations, or those of the publisher, the editors and the reviewers. Any product that may be evaluated in this article, or claim that may be made by its manufacturer, is not guaranteed or endorsed by the publisher.

## Supplementary Material

Supplementary Figure 1 The entire Sanger sequencing of the edited StLDP gene in primary exconjugant StLDP-KO (3). The minimized figure is presented in Figure 2D.

Supplementary Figure 2 Wt StLDP gene, amplified with INF-StLDP-F and INF-StLDP-R primers (Materials and Methods), produces a 1,400-bp product; the amplicon of ~700 bp in StLDP-KO (3) indicates a deletion resulting from the multiplexed targeting of all four sgRNA. The positions of DNA size markers are indicated.
