# Supplementary material for: Multiplexed Genome Editing via an RNA Polymerase II Promoter-Driven sgRNA Array in the Diatom Phaeodactylum tricornutum: Insights Into the Role of StLDP
Source: Front Plant Sci. 2022 Jan 4;12:784780. doi: 10.3389/fpls.2021.784780 (PMC8763850; doi:10.3389/fpls.2021.784780)
Supplement: Supplementary Figure 1 — The entire Sanger sequencing of the edited StLDP gene in primary exconjugant StLDP-KO (3). The minimized figure is presented in Figure 2D. [file data_sheet_1.pdf]

Supplementary material

Multiplexed Genome Editing via RNA Polymerase II Promoter Driven sgRNA Array in the diatom *Phaeodactylum tricornutum*: insights into the role of StLDP

Yogesh Taparia [1#], Achintya Kumar Dolui[1#], Sammy Boussiba[1] and Inna Khozin-Goldberg[1*]

Microalgal Biotechnology Laboratory, French Associates Institute for Agriculture & Biotechnology of Drylands, The Jacob Blaustein Institutes for Desert Research, Ben-Gurion University of the Negev, Midreshet Ben-Gurion, Israel, 849900. tapariayogesh@gmail.com; achintya86@gmail.com; boussiba@bgu.ac.il; khozin@bgu.ac.il

* Correspondence: khozin@bgu.ac.il

\# These authors contributed equally to this study

1. Nucleotide Sequences Level-0 parts for multiplex CRISPR/Cas9 Episome

pCA-L1-1-Episome-ΔShBle(CEN_ARS_HIS_OriT)

ATGggagctagctcgtcttcaGCGAGCATCACGTGCTATAAAAATAATTATAATTTAAATTTTTTAATATAAATAT
ATAAATTAAAAATAGAAAGTAAAAAAAGAAATTAAAGAAAAAATAGTTTTTGTTTTCCGAAGATGTA
AAAGACTCTAGGGGGATCGCCAACAAATACTACCTTTTATCTTGCTCGTCCTGCTCTCAGGTATTAATG
CCGAATTGTTTCATCTTGTCTGTGTAGAAGACCACACACGAAAATCCTGTGATTTTACATTTTACTTAT
CGTTAATCGAATGTATATCTATTTAATCTGCTTTTCTTGTCTAATAAATATATATGTAAAGTACGCTTTT
TGTTGAAATTTTTTTAAACCTTTGTTTATTTTTTTTTCTTCATTCCGTAACTCTTCTACCTTCTTTATTTACT
TTCTAAAATCCAAATACAAAACATAAAAATAAATAAACACAGAGTAAATTCCCAAATTATTCCATCAT
TAAAAGATACGAGGCGCGTGTAAGTTACAGGCAAGCGATCCTAGTACACTCTATATTTTTTTATGCCTC
GGTAATGATTTTCATTTTTTTTTTCCACCTAGCGGATGACTCTTTTTTTTTCTTAGCGATTGGCATTATCA
CATAATGAATTATACATTATATAAAGTAATGTGATTTCTTCGAAGAATATACTAAAAAATGAGCAGGC
AAGATAAACGAAGGCAAAGATGACAGAGCAGAAAGCCCTAGTAAAGCGTATTACAAATGAAACCAA
GATTCAGATTGCGATCTCTTTAAAGGGTGGTCCCCTAGCGATAGAGCACTCGATCTTCCCAGAAAAAG
AGGCAGAAGCAGTAGCAGAACAGGCCACACAATCGCAAGTGATTAACGTCCACACAGGTATAGGGTT
TCTGGACCATATGATACATGCTCTGGCCAAGCATTCCGGCTGGTCGCTAATCGTTGAGTGCATTGGTGA
CTTACACATAGACGACCATCACACCACTGAAGACTGCGGGATTGCTCTCGGTCAAGCTTTTAAAGAGG
CCCTAGGGGCCGTGCGTGGAGTAAAAAGGTTTGGATCAGGATTTGCGCCTTTGGATGAGGCACTTTCC
AGAGCGGTGGTAGATCTTTCGAACAGGCCGTACGCAGTTGTCGAACTTGGTTTGCAAAGGGAGAAAGT
AGGAGATCTCTCTTGCGAGATGATCCCGCATTTTCTTGAAAGCTTTGCAGAGGCTAGCAGAATTACCCT
CCACGTTGATTGTCTGCGAGGCAAGAATGATCATCACCGTAGTGAGAGTGCGTTCAAGGCTCTTGCGG
TTGCCATAAGAGAAGCCACCTCGCCCAATGGTACCAACGATGTTCCCTCCACCAAAGGTGTTCTTATGT
AGTTTTACACAGGAGTCtggacttgaccaatgGATCGTCTTGCCTTGCTCGTCGGTGATGTACTTCACCAGCTCC
GCGAAGTCGCcCTTCTTGATGGAGCGCATGGGGACGTGCTTGGCAATCACGCGCACCCCCCGGCCGTTT
TAGCGGCTAAAAAAGTCATGGCTCTGCCCTCGGGCGGACCACGCCCATCATGACCTTGCCAAGCTCGT
CCTGCTTCTCTTCGATCTTCGCCAGCAGGGCGAGGATCGTGGCATCACCGAACCGCGCCGTGCGCGGG
TCGTCGGTGAGCCAGAGTTTCAGCAGGCCGCCCAGGCGGCCCAGGTCGCCATTGATGCGGACCAGCTC
GCGGACGTGCTCATAGTCCACGACGCCCGTGATTTTGTAGCCCTGGCCGACGGCCAGCAGGTAGGCCG
ACAGGCTCATGCCGGCCGCCGCCGCCTTTTCCTCAATCGCCCTTCGTTCGTCTGGAAGGCAGTACACCT
TGATAGGTGGGCTGCCCTTCCTGGTTGGCTTGGTTTCATCAGCCATCCGCTTGCCCTCATCTGTTACGCC
GGCGGTAGCCGGCCAGCCTCGCAGAGCAGGATTCCCGTTGAGCACCGCCAGGTGCGAATAAGGACA
GTGAAGAAGGAACACCCGCTCGCGGGTGGGCCTACTTCACCTATCCTGCCCGGCTGACGCCGTTGGAT
ACACCAAGGAAAGTCTACACGAACCCTTTGGCAAAATCCTGTATATCGTGCGAAAAAGGATGGATATA
CCGAAAAAATCGCTATAATGACCCCGAAGCAGGGTTATGCAGCGGAAGATGCTTaccatgattacgccaagctcga
aattaaccctcactaaagggaacaaaagctggtacctaacaggattagtgcaattcgagttgaatcactgggaaaaacattgtcttctttttttatattatcatttgcattagtgct

gcagtcgtagatacttgttggttgaaagacatcagcctcggagatctgggcccatgcggccgcaacaactacctcgactttggctgggacactttcagtgaggacaagaa
gcttcagaagcgtgctatcgaactcaaccagggacgtgcggcacaaatgggcatccttgctctcatggtgcacgaacagttgggagtctctatccttccttaaaaatttaatt
ttcattagttgcagtcactccgctttggtttcacagtcaggaataacactagctcgtcttcaatcacgtgccgctGCA

Nucleotide sequences of Level-0 of parts and fragments used to assemble pCA-L1-2-sgRNA-Array

>pL0-AC-PrH4-1b
GGAGattgttggcgtactgctgatgctgctggtgtgttgctgtggtggttgcggttccggaaggcggcaatgcctgcgacgccgtggtaaagctggtttcggaagcatc
gtcgggagggtagaaatgtcccccgaagcagcagcgcgtgccgcttctcgcccttcaaagtcttctaacgtggagttcccgtgattgtggttaccgttctgggcattgcc
ggatccccgttgaaagacggctcccatccttcgcgacccggggcgttttgcgtgagtcgctggtgccgaggccgtgcgctggtgttcctcatagtcttccggtgtagctcc
cgccgggagtgaagcttcttgccccatgcgactacgaatatgtagacgaaattcggttgctgcttccgattacacgggagagaacaaagtccccgtcagcaaaccctatc
gacttatcaagatgctgcactcgtttgaaggaagccctcgtcaaactaacagtgactgccaagcaatttctgtgaaacccgacccggtacaacaatcaaagattatgcaca
cacgaacactaatagtagacaagtcttctgcaaatcctcactacaaggggaacgactaaagttggtcgtccatcgttagcgtcgtcacgaacgcatcgagcgaggcgga
gaaaacgggagctgcggcagccgatgccctgccgtcggactctttcccgggaaacgggtacactcctccgcgccaacaacatattactactactaccaagaacgtccac
ggccttgtcgtgcgttacgctcgtccaacgcgtgcggggtaaataatgtctcggtttgctaagtagcgcacagctaaatagatgaccgttattgtatttaagatcattcaatatt
gattgcattgtactttgcgtcaaactgaaattcccttgtactaacggttaacccgtcaaccctaagcgttcgcccaaagtagtcaaccgggacacgcgaaccgacattgggc
agatctttcacagacagaaaaccatttccaatccaaataagcatgactattacacacccattcgtagcgcgaggacaaactgacagctccaacaaaatgcgccaacatcgt
acattgtaagaagcttacggaacactatgtatgtagaaccatacgaacagcaactagtactggccatcgagcagcggtgactcccggctttcgaagcgctgtgaaggtta
cactctcacaattcgctctcggcagtaaccgacaatcgttttactcaataccgaaaacaaacaacagccaacAATG

>pL0-CD-HH_Ribozyme (underlined sequences are reverse complement and palindromic to each other)
AATGaccttaCTGATGAGTCCGTGAGGACGAAACGAGTAAGCTCGTCtaAGGT

In the following sgRNA-1 to 4 sequences BsaI, uLoop Overhangs, 20bp target and gRNA for SpCas9 are indicated as highlight or color scheme. These dsDNA fragments were assembled by amplifying two 80 bp oligonucleotide primers with <u>complimentary sequences</u>. Additional AA/TT sequences (total 6 to 8 bp including 4bp Golden Gate sequence) should be added 5’ to BsaI sites to ensure efficient digestion of fragments. The reverse primers in each case can be reused.

>sgRNA-1
aaggtctcaAGGTGCGACTGGGTAAGTCGTACGGTTTTAGAGCTAGAAATAGCAAGTTAAAATAAGGCTAG
TCCGTTATCAACTTGAAAAAGTGGCACCGAGTCGGTGCTTTTttTTCGtgagacctt

>sgRNA-2
aaggtctcaTTCGTCTGGCACTACAGCGGACGAGTTTTAGAGCTAGAAATAGCAAGTTAAAATAAGGCTAG
TCCGTTATCAACTTGAAAAAGTGGCACCGAGTCGGTGCTTTTttTCCAtgagacctt

>sgRNA-3
aaggtctcaTCCATTGCCATTTTCAATTCACAAGTTTTAGAGCTAGAAATAGCAAGTTAAAATAAGGCTAGT
CCGTTATCAACTTGAAAAAGTGGCACCGAGTCGGTGCTTTTttGAGAtgagacctt

>sgRNA-4
aaggtctcaGAGATTGTACGCGAGCACGAACGCGTTTTAGAGCTAGAAATAGCAAGTTAAAATAAGGCTAG
TCCGTTATCAACTTGAAAAAGTGGCACCGAGTCGGTGCTTTTttATAAtgagacctt

>pL0-4E-HDV
ATAAggccggcatggtcccagcctcctcgctggcgccggctgggcaacatgcttcggcatggcgaatgggacGCTT

>pL0-EF-TrFcpA
GCTTcagaagcgtgctatcgaactcaaccagggacgtgcggcacaaatgggcatccttgctctcatggtgcacgaacagttgggagtctctatccttccttaaaaattta
attttcattagttgcagtcactccgctttggtttcacagtcaggaataacactagctcgtcttcaccatggatgccaatctcgcctattcatggtgtataaaagttcaacatccaa
agctagaacttttggaaagagaaagaatatccgaatagggcacggcgtgccgtattgttggagtggactagcagaaagtgaggaaggcacaggatgagttttctcgaC
GCT

Sequences of Level-0 of parts used to assemble pCA-L1-3-SpCas9(PX459)-P2A-ShBle/Bsr1

>pL0-AC-Pr49202
GGAGtccaaacggtacgtcagatcccacgtcactaccgcacccggttattccagtgcgtaagctcgtagtggaagaacaggctgcggtagtgacgtggaatttgaca
ctttccaatcttcaagacctctagccagcaaagacttcttgaatctgcaaggatattctatttagatataagtatatacttgacagtgaggggggtcgtgaccgtctctcgctgg
gatagagaagatggcaaccattgaattgagaactcgaaacttgagcttggttggatcttgatcattctgaatttgcaggataggttaacagtaaaagtcaatcttgcgctcgc
catctaccagaaagcacgtgggtgcccagatcctcccaacgagcgaacggacggatgctagtcggggggatctctgtgggccattcaaacgaaaggcagatggcatc
gttccaagatctattggtcattgggcttctcatacagtcattgacaccacgattgaagacaaatttttgttgacagtgaaatcgttgcggacgcgctccgattcaaaagtttccc
tactcggtcgcgtcaaccgaccttcccctcgtgagtttacagcttgatacgccctatttggcttcctatgtaaagtctccagtgatcatattaaagtcattggtcatgacactctc
cttgttcaaaatttggtaccatacaatgcgcggagcagtcagttctcatttccagcgatcgttaaaacaattagagaaatgccttacttctctctttatatctgtattactcagagt
cagtgagtagtacgaatttccaacgtcctagctccaatttatggtttcaaacaatccgaattcgttggtccacagtcaaggaattcgcaagaatccctatttcactaggcgtgc
actatggggtcgcttttgaggttctcatttgcggcgatcgttaaaacgattacagaaatgctacacttcgctctttatatatattactcattgagcatcaggaatgtccaatgtcgt
aactccgattcatggtttcaaacaatccgaattcgttggtccgaggaatccgcaaaaagtaattgtagtctatgtacgaccgagtcgctggtgagggtttttttgtcgttggattg
ctcgagcgaattctccgagattgtacagaaagctcaactttcggaaggcatctatgtggtccaaccaatctattcaaatatatcaattgttaagagcagagttcgatcctcact
atgtggaggggcatggtgagattacggagtgccgagacataagagctccctgcaatcaagttgatggacatgcttcgaaaatgccatgactgagaacgagaacgccca
gatgctccgcggcacgagaactcgatgaacacgaaccgaatccaacgccaaaaacgcgttgtccaaaacattgcaactctctctgataaaaaccaaaacgccgcaaatt
gcaccagcttttttggttgtcctacgcctctttccgcaccaccAATG

>pL0-CD-SpCas9(PX459)_3xFLAG_SV40-NLS_Nucleoplasmin-NLS
AATGgactataaggaccacgacggagactacaaggatcatgatattgattacaaagacgatgacgataagatggccccaaagaagaagcggaaggtcggtatcca
cggagtcccagcagccgacaagaagtacagcatcggcctggacatcggcaccaactctgtgggctgggccgtgatcaccgacgagtacaaggtgcccagcaagaaa
ttcaaggtgctgggcaacaccgaccggcacagcatcaagaagaacctgatcggagccctgctgttcgacagcggcgaaacagccgaggccacccggctgaagaga
accgccagaagaagatacaccagacggaagaaccggatctgctatctgcaagagatcttcagcaacgagatggccaaggtggacgacagcttcttccacagactgga
agagtccttcctggtggaagaggataagaagcacgagcggcaccccatcttcggcaacatcgtggacgaggtggcctaccacgagaagtaccccaccatctaccacct
gagaaagaaactggtggacagcaccgacaaggccgacctgcggctgatctatctggccctggcccacatgatcaagttccggggccacttcctgatcgagggcgacct
gaaccccgacaacagcgacgtggacaagctgttcatccagctggtgcagacctacaaccagctgttcgaggaaaaccccatcaacgccagcggcgtggacgccaag
gccatcctgtctgccagactgagcaagagcagacggctggaaaatctgatcgcccagctgcccggcgagaagaagaatggcctgttcggaaacctgattgccctgagc
ctgggcctgacccccaacttcaagagcaacttcgacctggccgaggatgccaaactgcagctgagcaaggacacctacgacgacgacctggacaacctgctggccca
gatcggcgaccagtacgccgacctgtttctggccgccaagaacctgtccgacgccatcctgctgagcgacatcctgagagtgaacaccgagatcaccaaggcccccct
gagcgcctctatgatcaagagatacgacgagcaccaccaggacctgaccctgctgaaagctctcgtgcggcagcagctgcctgagaagtacaaagagattttcttcgac
cagagcaagaacggctacgccggctacattgacggcggagccagccaggaagagttctacaagttcatcaagcccatcctggaaaagatggacggcaccgaggaac
tgctcgtgaagctgaacagagaggacctgctgcggaagcagcggaccttcgacaacggcagcatcccccaccagatccacctgggagagctgcacgccattctgcgg
cggcaggaagatttttacccattcctgaaggacaaccgggaaaagatcgagaagatcctgaccttccgcatcccctactacgtgggccctctggccagggggaaacagca
gattcgcctggatgaccagaaagagcgaggaaaccatcaccccctggaacttcgaggaagtggtggacaagggcgcttccgcccagagcttcatcgagcggatgacc
aacttcgataagaacctgcccaacgagaaggtgctgcccaagcacagcctgctgtacgagtacttcaccgtgtataacgagctgaccaaagtgaaatacgtgaccgagg
gaatgagaaagcccgccttcctgagcggcgagcagaaaaaggccatcgtggacctgctgttcaagaccaaccggaaagtgaccgtgaagcagctgaaagaggacta
cttcaagaaaatcgagtgcttcgactccgtggaaatctccggcgtggaagatcggttcaacgcctccctgggcacataccacgatctgctgaaaattatcaaggacaagg
acttcctggacaatgaggaaaacgaggacattctggaagatatcgtgctgaccctgacactgtttgaggacagagagatgatcgaggaacggctgaaaacctatgccca
cctgttcgacgacaaagtgatgaagcagctgaagcggcggagatacaccggctggggcaggctgagccggaagctgatcaacggcatccgggacaagcagtccgg
caagacaatcctggatttcctgaagtccgacggcttcgccaacagaaacttcatgcagctgatccacgacgacagcctgacctttaaagaggacatccagaaagcccag
gtgtccggccagggcgatagcctgcacgagcacattgccaatctggccggcagccccgccattaagaagggcatcctgcagacagtgaaggtggtggacgagctcgt
gaaagtgatgggccggcacaagcccgagaacatcgtgatcgaaatggccagagagaaccagaccacccagaagggacagaagaacagccgcgagagaatgaag
cggatcgaagagggcatcaaagagctgggcagccagatcctgaaagaacaccccgtggaaaacacccagctgcagaacgagaagctgtacctgtactacctgcaga
atgggcgggatatgtacgtggaccaggaactggacatcaaccggctgtccgactacgatgtggaccatatcgtgcctcagagctttctgaaggacgactccatcgacaa
caaggtgctgaccagaagcgacaagaaccggggcaagagcgacaacgtgccctccgaagaggtcgtgaagaagatgaagaactactggcggcagctgctgaacgc
caagctgattacccagagaaagttcgacaatctgaccaaggccgagagaggcggcctgagcgaactggataaggccggcttcatcaagagacagctggtggaaaccc
ggcagatcacaaagcacgtggcacagatcctggactcccggatgaacactaagtacgacgagaatgacaagctgatccgggaagtgaaagtgatcaccctgaagtcc
aagctggtgtccgatttccggaaggatttccagttttacaaagtgcgcgagatcaacaactaccaccacgcccacgacgcctacctgaacgccgtcgtgggaaccgccct
gatcaaaaagtaccctaagctggaaagcgagttcgtgtacggcgactacaaggtgtacgacgtgcggaagatgatcgccaagagcgagcaggaaatcggcaaggcta
ccgccaagtacttcttctacagcaacatcatgaactttttcaagaccgagattaccctggccaacggcgagatccggaagcggcctctgatcgagacaaacggcgaaac
cggggagatcgtgtgggataagggccgggattttgccaccgtgcggaaagtgctgagcatgccccaagtgaatatcgtgaaaaagaccgaggtgcagacaggcggct
tcagcaaagagtctatcctgcccaagaggaacagcgataagctgatcgccagaaagaaggactgggaccctaagaagtacggcggcttcgacagccccaccgtggc
ctattctgtgctggtggtggccaaagtggaaaagggcaagtccaagaaactgaagagtgtgaaagagctgctggggatcaccatcatggaaagaagcagcttcgagaa
gaatcccatcgactttctggaagccaagggctacaaagaagtgaaaaaggacctgatcatcaagctgcctaagtactccctgttcgagctggaaaacggccggaagaga
atgctggcctctgccggcgaactgcagaagggaaacgaactggccctgccctccaaatatgtgaacttcctgtacctggccagccactatgagaagctgaagggctccc
ccgaggataatgagcagaaacagctgtttgtggaacagcacaagcactacctggacgagatcatcgagcagatcagcgagttctccaagagagtgatcctggccgacg
ctaatctggacaaagtgctgtccgcctacaacaagcaccgggataagcccatcagagagcaggccgagaatatcatccacctgtttaccctgaccaatctgggagcccc
tgccgccttcaagtactttgacaccaccatcgaccggaagaggtacaccagcaccaaagaggtgctggacgccaccctgatccaccagagcatcaccggcctgtacga
gacacggatcgacctgtctcagctgggaggcgacaaaaggccggcggccacgaaaaaggccggccaggcaaaaaagaaaaaggcAGGT

>pL0-DB5-P2A
AGGTgcccgcgagacgggaagcggagctactaacttcagcctgctgaagcaggctggcgacgtggaggagaaccctggacctggactccggcgTTGC

>pL0-B5E-ShBle
TTGCatggccaagttgaccagtgccgttccggtgctcaccgcgcgcgacgtcgccggagcggtcgagttctggaccgaccggctcgggttctcccgggacttcgtg
gaggacgacttcgccggtgtggtccgggacgacgtgaccctgttcatcagcgcggtccaggaccaggtggtgccggacaacaccctggcctgggtgtgggtgcgcg
gcctggacgagctgtacgccgagtggtcggaggtcgtgtccacgaacttccgggacgcctccgggccggccatgaccgagatcggcgagcagccgtgggggcggg
agttcgccctgcgcgacccggccggcaactgcgtgcacttcgtggccgaggagcaggactgaGCTT

>pL0-B5E-Bsr1
TTGCatgaaaacgtttaatatctcgcaacaagaccttgaacttgtcgaagtcgcaacggaaaaaattacgatgctttacgaagacaataaacatcatgtcggagcagca
attcgaacgaaaacgggagaaattatttcggcagtccatattgaagcatacattggacgagtcacggtctgcgcagaagcaattgcaattggatcggcagtctcgaatgg
acaaaaagactttgacacgattgtcgcagtccgacatccatactcggacgaagtcgatcgatcgattcgagtcgtctcgccatgcggaatgtgccgagaacttatttcgga
ctacgcaccagactgctttgtccttattgaaatgaatggaaaacttgtcaaaacgacgattgaagaacttattccacttaaatacacgcgaaattgaGCTT

>pL0-EF-Tr49202
GCTTttcgatcgacgagcttaccttcacggccaccgttcccgaccccgacgttgttcaggcgatcattacgggtggcagcgccgctccgggagccaccggtttgtac
ggcattgccaccgcagatgccgagtgcccggccaacgagtgcatccatgtggacgtgattgtgtacgccattttcgcgaatgaagccacgaccaacttgattgtggaaat
cagcggcgacgcggtcatgtctgtgggacgtactgcccgcaagttgcgtgcgaaagtccagcccacacgcgagttgcaggagacgctccagatgcaagcctttcgtaa
cgagattgtcctcccccctttgcctggttcgggggccacgttggccaacgtgtgcgcggcggcggtgcccttcgccgtggccgtggtggccggtgcgctcgctctttaaa
aagtgagagtttgtgtgtgtgaaagacgcgtcgcgagacgctccttatttccgtcggcatgtgtcttgtcgtggtcctatgtgatccccgggtcggttcacgggagttgccg
aagtagagaataagaattatataactgtacagagatacagacatgtgctgctattacgtccaacagtaggtatacacctcccgggcaatagagtgggccacatccatacac
acacacccgagttggtagattgatttcggttcgtcgaatgccaagaatgtgcttcctgaagaaaatttgctccttcccactccgtccgtacgaccatccgcccgtgtacagcg
cggcagaagtagaaacgagtaccaaggctctgtgtgtgtgtgtgtaccgtttggacgcgtcggcaaaggtacggcagtcctccgggtgtgtcccgtactcgtggtaccaa
cgaatctgcacaattgtgccgttcggaggacacgccttcccggatgcatgcatccggaagaccgtcgatgcgattCGCT

>pCA-L1-4-Spacer20bp
CAGaggagATCGCGCCTCTAGTAAGGTTcgctaGGT

Supplementary Figure 1 The entire Sanger sequencing of the edited StLDP gene in primary exconjugant *StLDP*-KO (3). The minimized figure is presented in Figure 2D. This Figure is provided as a separate file.

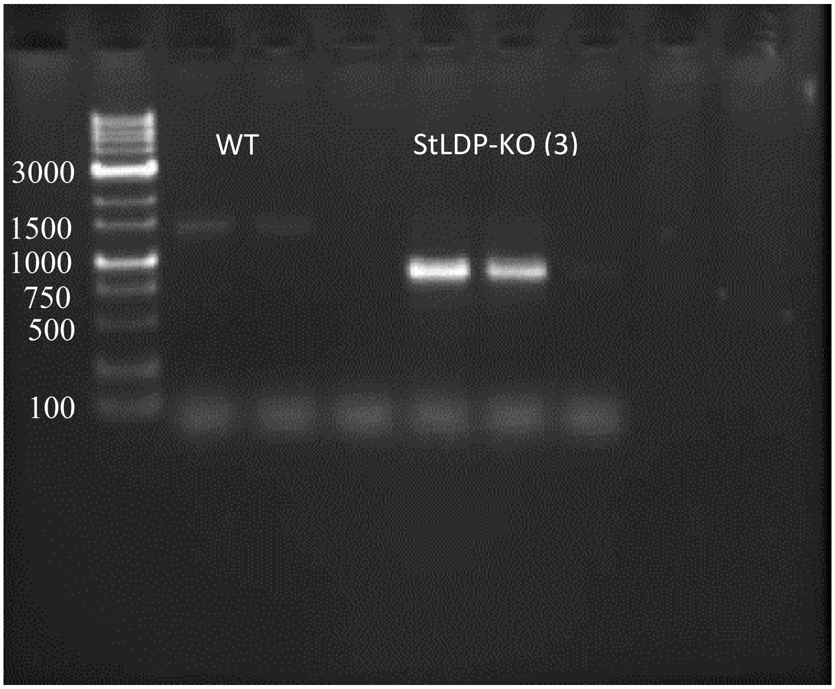

Supplementary Figure 2. Wt *StLDP*, amplified with INF-StLDP-F and INF-StLDP-R primers (Materials and Methods), produces a 1400-bp product (lanes 2 and 3); an amplicon in StLDP-KO (3) indicates a deletion resulting from the multiplexed targeting of all four sgRNA. The positions of DNA size markers (in bp) are indicated.

Lane 1 – 1kb ladder (MD015-R500)-Bio-Helix
Lanes 2, 3, 4 – WT Pt1 (annealing temperature 62˚C, 65˚C, 68˚C)
Lanes 5, 6, 7 – StLDP-KO (3) (62˚C, 65˚C, 68˚C)

2. Supplementary methods

Improved Lysis Buffer for Diatom On-Colony PCR
Protocol from Nymark et al., 2016 (www.bio-protocol.org/e2442) with some modifications*

Lysis Buffer (6x)

Triton-X 100	100 µL	(final concentration 1%)
50mM Tris-HCl (pH = 8)	4 mL	(final concentration 20 mM)
0.5M EDTA	40 µL	(final concentration 2 mM)
PVP K25*	360 mg	
Final volume	10 mL	
Post Autoclave		
β-mercaptoethanol*	120 µL	

Lysis buffer can be stored at room temperature for ~ 1 year.

Prior to on-colony PCR from solid media grown colonies
Pick stable transgenic colonies using sterile 10 µL tips and patch them on to solid 1/2xRSE +5% LB medium. Sufficient diatom biomass grows within 4-5 days.

Directions for on-colony PCR from solid media patched colonies:

1. From the selection plates directly pick colonies using sterile 10 µL tips into 10µL medium in a sterile PCR plate well. Agitate the plate to get rid of all the colony material.
2. Patch 3 µL (do not put more) of this *Phaeodactylum* suspension onto fresh selection plate and allow to grow for 3-5 days. Also patch WT as a control. When sufficient growth is seen (by day 3-5), colony PCR can be performed.
3. Using a sterile 10 µL tip pick sufficient material (do not take excess material as it inhibits PCR) and drop into a PCR plate with 10 µL of lysis buffer (put the PCR plate with buffer on ice while picking spotted colonies). Agitate the PCR plate to resuspend the material in lysis buffer and place back on ice for 15 minutes. Seal the plate.
4. On a thermocycler heat the picked colonies for 10 min at 95°C. If PCR screening will be performed later, save the material in -20°C without diluting the material.
5. At the time of performing PCR add 50 µL ddH20 (1:5 dilution) to each sample and use 1.5 µL of this diluted lysed cell as a template in a 10 µL PCR reaction (40 cycles). Throughout the setup of PCR keep the lysed template on ice.
6. Save the diluted lysed template from step 6 indefinitely in -20°C. When using diluted template from -20°C, thoroughly resuspend the solution before adding to PCR mix.

Important Notes:

1. PCR screening from patched plates is reliable, fast and convenient method for screening (than liquid culture based cPCR).
2. RSE Liquid medium grown cells should be avoided due to additional steps in removal of salt from the cell pellet. Additionally, colonies picked into deep well plates grow slowly and have to be agitated with pipet tip to prevent them from adhering to bottom of the deep-well plate regularly.
3. Use of half-strength salt medium for culturing (solid/liquid medium) is preferable for PCR screening.
4. Adding excess template to the PCR reaction inhibits successful amplification.
5. PCR thermocycling for 40 cycles ensures amplification even in the case of very few cells.
6. Lysed template stored at -20°C can be reused for colony PCR even after 1 year.
7. PVP K25 (water-soluble PVP) and β-mercaptoethanol were added to alleviate PCR inhibition by secondary metabolites and allow PCR from old-patched plates.
